# Editorial: Multisensory integration: unveiling the complexities of perception

**DOI:** 10.3389/fnhum.2026.1959500

**Published:** 2026-09-25

**Authors:** Alessandro Bortolotti, Baingio Pinna, Nicola Di Stefano, Caterina Padulo

**Affiliations:** 1 University of Studies G. d'Annunzio Chieti and Pescara, Chieti, Italy; 2 University of Sassari, Sassari, Italy; 3 National Research Council (CNR), Rome, Italy; 4 University of Naples Federico II, Naples, Italy

**Keywords:** cognition, complexity, multisensory, perception, sensory integration

## Introduction

Every moment of waking life confronts the nervous system with a torrent of signals arriving simultaneously through eyes, ears, skin, nose, and the body's internal receptors. Making sense of such a multiplicity of inputs requires more than processing each channel in isolation: the brain must decide which signals belong together, weigh their relative reliability, and merge them into a single, actionable representation of the world. This is the work of multisensory integration, classically defined as the neural process by which information from different sensory modalities is combined to enhance the salience and reliability of the resulting percept (Stein and Meredith, 1993). Once thought to be confined to late, associative stages of a strictly hierarchical processing chain, multisensory interactions are now known to occur at multiple levels of the nervous system, from subcortical structures such as the superior colliculus to primary sensory cortices themselves (Driver and Noesselt, 2008).

Multisensory integration is not simply additive: it involves flexible, context-dependent computations that can enhance, suppress, or reweight individual sensory inputs. A large body of psychophysical work has shown that this reweighting closely approximates statistically optimal cue combination, in which each sense is weighted according to its momentary reliability (Ernst and Banks, 2002; Alais and Burr, 2004). Phenomena such as the McGurk effect, in which lip movements alter the perceived identity of speech sounds (McGurk and MacDonald, 1976), and the sound-induced flash illusion (Shams et al., 2000), illustrate how profoundly cross-modal interactions can shape, and distort, conscious experience. Attention and top-down expectation further modulate when and how strongly these interactions occur (Talsma et al., 2010), while systematic crossmodal correspondences between, for instance, pitch and visual size or brightness reveal that the mapping between senses is far from arbitrary (Spence, 2011) a principle whose reach extends well beyond the laboratory, into applied domains such as consumer neuroscience (Bortolotti, 2025). The full expression of these integrative abilities is not present at birth but unfolds gradually over childhood, a protracted developmental trajectory that may offer a window of opportunity for early intervention in atypical sensory processing (Bremner et al., 2012).

This Research Topic, “*Multisensory integration: unveiling the complexities of perception*,” was launched to bring together contributions that probe this flexibility from complementary angles: computational modeling of how signals are combined, psychophysical and electrophysiological investigation of cross-modal effects on perception and action, and applied work exploring how multisensory principles play out in naturalistic and developmental contexts. The Research Topic brings together twelve published articles and two further contributions that have been accepted and are currently in production, for a total of fourteen contributions, spanning Frontiers in Human Neuroscience, Frontiers in Neuroscience, and Frontiers in Psychology, offering a timely snapshot of a field that continues to challenge the classical, hierarchical view of sensory processing.

## Computational principles of sensory combination

A recurring theme is the search for the computational rules that govern how the brain weighs and merges different sources of information. Billock et al. show that multisensory suppressive neurons, rather than being a mere nuisance for integration models, can implement a Bayesian-like, nonlinearly weighted combination of sensory inputs, offering a neurally grounded account of how statistically optimal integration might be instantiated at the single-cell level. Complementary evidence comes from touch: Allen and Goldreich demonstrate that humans optimally combine cutaneous and proprioceptive cues when judging object size, extending the logic of statistically optimal fusion beyond the classical audiovisual domain into haptic perception. At the interface between vision and touch, Umemura and Iwaki reveal that the perceived simultaneity of visual and haptic events is not fixed but is shaped by the contextual consistency of the stimuli, underscoring how integration windows are recalibrated online rather than hard-wired.

## Cross-modal influences on perception, attention, and action

A second group of articles examines how the information gathered from one modality shapes processing in another, and how these interactions unfold in time. Rizzuto et al. show that fixational eye movements, typically studied as a purely visual phenomenon, track the segmentation of auditory streams, providing a striking example of oculomotor signatures of auditory perceptual organization. In the domain of social perception, Toro et al. combine behavioral and ERP measures to demonstrate that contextual cues systematically shape facial emotion recognition, indicating that emotion perception is inherently an integrative, context-sensitive process rather than a modular readout of facial configuration alone. Moving to sensorimotor coupling, Zhao et al. report that the well-known action effect on perception is not modulated by cognitive load, suggesting a degree of automaticity and resource-independence in how action shapes perceptual outcomes. Finally, Adamski and Latka revisit the classic rod-and-frame illusion using virtual reality, offering a methodological update that reinterprets a decades-old visuo-vestibular phenomenon with modern immersive tools.

## Multisensory integration in ecological, developmental, and applied contexts

Beyond laboratory psychophysics, several contributions situate multisensory integration within real-world or developmentally relevant settings. Huo et al. investigate how the duration and concentration of olfactory stimuli affect sleep-related arousal during fatigue driving, pointing to concrete safety applications for multisensory countermeasures against drowsiness. Shafique et al. ask whether spatialized audio enhances the construction of mental representations of space, a question with direct relevance for assistive technology and sensory-substitution devices. Sun et al. examine perception-action coupling during discriminative interceptive actions, clarifying how perceptual decisions and motor execution are jointly tuned in time-pressured tasks. On the developmental and clinical side, Ge et al. show that structured basketball training can improve sensory integration in Chinese children with moderate sensory integration challenges, offering encouraging evidence for embodied, play-based interventions.

## Toward an integrative and interdisciplinary framework

Closing the Research Topic on a more theoretical note, Bortolotti et al. introduce the notion of supercomplexity to bridge aesthetics and cognition, proposing a conceptual framework in which the integration of multiple, sometimes conflicting, perceptual and cognitive dimensions underlies aesthetic experience itself. This contribution reminds us that multisensory integration is not confined to the combination of low-level sensory estimates but extends into higher-order domains such as value, meaning, and aesthetic appreciation.

At the time of writing, the Research Topic continues to grow: an original research contribution by Chen et al., examining how environmental cues shape human face-value perceptions, was accepted for publication in Frontiers in Psychology on 14 April 2026, and a further original research contribution by He and Zeng (in press), on visual perception and information design in metro wayfinding systems, was accepted for publication in Frontiers in Psychology on 10 February 2026; both are currently in production within this Research Topic. We look forward to these additional papers, which will further extend the scope of the Research Topic, and we will update our characterization of the Topic, including full bibliographic details, once their final versions are published.

Taken together, the fourteen contributions in this Research Topic, 12 already published and two accepted and in production, llustrate that multisensory integration cannot be captured by a single mechanism or a single level of description. Optimal-cue-combination models, single-neuron nonlinearities, oculomotor and electrophysiological markers, developmental interventions, and theoretical frameworks bridging perception and aesthetics all contribute pieces of the same puzzle. The diversity of methods, computational modeling, psychophysics, ERP, virtual reality, and applied field studies, mirrors the diversity of contexts in which multisensory integration operates, from milliseconds-long neural computations to years-long developmental trajectories.

Several open questions emerge from this body of work and point to promising directions for future research. How do the statistically optimal weighting principles identified in controlled psychophysical settings scale up to the noisy, dynamic conditions of everyday life, such as driving or social interaction? To what extent are the automaticity and resistance to cognitive load reported for some cross-modal effects a general property of multisensory processing, or are they specific to particular tasks and modalities? And how can insights from developmental interventions and technological applications, such as spatialized audio and virtual reality, be more systematically integrated with the computational and neural models that currently dominate the basic-science literature?

We believe that answering these questions will require continued interdisciplinary dialogue among computational neuroscientists, psychophysicists, clinicians, and applied researchers, precisely the kind of dialogue this Research Topic was designed to foster. We thank all the authors for their valuable contributions, the reviewers for their rigorous and constructive evaluations, and the Frontiers editorial team for their support throughout the process.
